# Different role of the supplementary motor area and the insula between musicians and non-musicians in a controlled musical creativity task

**DOI:** 10.1038/s41598-019-49405-5

**Published:** 2019-09-10

**Authors:** Marcella Pereira Barbosa de Aquino, Juan Verdejo-Román, Miguel Pérez-García, Purificación Pérez-García

**Affiliations:** 1 Faculty of Education Sciences at the University of Granada, Granada, Spain; 2 Mind, Brain, and Behavior Research Center (CIMCYC), University of Granada, Granada, Spain; 3 Laboratory of Cognitive and Computational Neuroscience (UCM-UPM), Center for Biomedical Technology (CTB), Pozuelo de Alarcón, Spain

**Keywords:** Neuroscience, Sensory processing

## Abstract

The ability to compose creative musical ideas depends on the cooperation of brain mechanisms involved in multiple processes, including controlled creative cognition, which is a type of creativity that has so far been poorly researched. Therefore, the objective of this study was to examine the brain evoked activations by using fMRI, in both musicians and non-musicians, during a general task of controlled musical creativity and its relationship with general creativity. Results revealed that during a rhythmic improvisation task, musicians show greater activation of the motor supplementary area, the anterior cingulate cortex, the dorsolateral prefrontal cortex, and the insula, along with greater deactivation of the default mode network in comparison with non-musicians. For the group of musicians, we also found a positive correlation between the time improvising and the activation of the supplementary motor area, whilst in the non-musicians group improvisation time correlated with the activation of the insula. The results found for the musicians support the notion that the supplementary motor area plays a role in the representation and execution of musical behaviour, while the results in non-musicians reveal the role of the insula in the processing of novel musical information.

## Introduction

Creativity is a unique feature of human behaviour, and is regarded as a fundamental activity in information processing^1^. Nonetheless, its neuronal substrates have received relatively little attention. The literature proposes that creativity or creative cognition results from a set of mental processes that associate, analyse, and interpret acquired knowledge to generate or develop a new, original, and useful product or idea^2–6^. This requires flexibility and cognitive persistence^7,8^ with the help of cognitive or emotional search processes^9^ or significant information^10^. In order for creativity to occur, it is necessary to use acquired knowledge^11,12^, memory^10^, normal reasoning and language^13^, as well as a cyclical process of the generation and evaluation of ideas^14^. In addition, the involvement of other cognitive processes has been proposed^15^ including the ability to allocate attentional resources for action^10,16^, the generation of new responses^17,18^ and the inhibition of repetitive responses^19^. The combination of these processes generates two types of creativity: one that appears spontaneously and unconsciously, and another that is a deliberate or controlled type of creativity, which is set in motion by a conscious effort based on prior knowledge or experiences along with the centralization of feelings and/or emotions as a tool for solving problems^20–22^. It therefore appears that creativity is a complex human process involving multiple functions.

From a cerebral point of view, the ability to compose creative ideas depends on the cooperation of brain mechanisms involved in the neurocognitive processes described above. Studies in creative people, using functional magnetic resonance imaging (fMRI), have systematically found a higher activation of the inferior frontal gyrus, with greater functional connectivity between this zone and regions of the default mode network (DMN), as well as stronger connections with the bilateral inferior parietal cortex and the left dorsolateral prefrontal cortex^23^. Moreover, more recent studies have focused on investigating functional connectivity as a way to understand what occurs within the dynamic interactions of the brain networks (e.g., default and executive control) when the brain creates and improvises^15^. In the case of spontaneous or unconscious creativity, a decrease in the influence of the dorsal prefontal cortex(dlPFC) on the creative process is observed^20^. Due to the relationship between creativity and self-referential thinking, some studies have suggested a role for the DMN (the medial and posterior parietal area) in the process of spontaneous creative cognition^23–25^. The parietal cortex, like the angular gyrus, has also been linked to spontaneous creativity^26^. In the case of deliberate or controlled creativity, the predominantly active cerebral structure is the prefrontal cortex^20,26^, with special attention being given to the dlPFC^20^, with the inferior parietal cortex being part of the control network that requires externally directed attention^15^ along with the dorsal anterior cingulate cortex (dACC)^27^. The dlPFC and the dACC are the structures responsible for the displacement of the mental set oriented towards the search for relevant information, the combination of elements in the semantic networks^20^, inhibitory control and updating of working memory, and action planning^28^. This network contributes to the evaluative mode of creative thinking, and plays a role in the analytical and deliberate processing of information, and consideration of the value of novel ideas^29^.

Music is a type of human activity that is characterized by high creativity. Webster^30^ defined creativity in music as: “the engagement of the mind in the active, structured process of thinking in sound for the purpose of producing some product that is new for the creator”. Therefore, the two main examples of musical creativity are the process of composing music, and musical improvisation. Other researchers have also considered musical improvisation as an expression of musical creativity because the fundamental element of improvisation is the creation of new music^31^. Furthermore, recent works in the neuroscience of creativity conceptualize musical improvisation as a real-time musical creative process in the brain^3^. When we observe the brain activity evoked by musical creativity, a set of prefrontal brain regions appear to be systematically activated, including the supplementary motor area (SMA), the medial prefrontal cortex, the left inferior frontal gyrus, the dlPFC and the dorsal premotor cortex^32^. More recently, musical creativity has been linked to structural changes in the brain, where people scoring higher on a musical creativity test showed a greater volume in the dorsomedial prefrontal cortex, the temporal cortex, the orbitofrontal cortex, and the amygdala^1^ all of which are associated with higher scores on a test of musical creativity. Further, in creative people there is an increase in grey matter in the left precuneus and cuneus^33^, whilst a positive correlation has been found between the composite creativity index scores and the cortical volume in the posterior cingulate cortex, an area that is involved in the formation and regulation of emotions and data processing related to behaviour, learning, and memory^34^. Thus, musically creative people show greater activation and cortical surface area or volume in motor-associative regions of higher cognitive order and domain-specific sound processing (dorsal premotor cortex, supplementary and pre-supplementary motor areas and the planum temporale), in regions related to the DMN (dorsomedial prefrontal cortex, middle temporal gyrus, and temporal pole) and in emotion-related regions i.e. the orbitofrontal cortex, temporal pole, and amygdala^1^.

In relation to the types of creativity described above, studies examining the neural correlates of musical activity have also used tasks of spontaneous creativity and controlled creativity. In the case of spontaneous creativity, the tasks require the participants to improvise without attending to a model, using only the help of spontaneous and implicit recombinations of their experience, their representations, and established routines^28^. One such example can be found in a study of improvisation in jazz pianists^35^, which found a dissociative pattern of activation of the prefrontal cortex, deactivating areas such as the dorsolateral prefrontal and lateral orbital cortex whilst activating the medial prefrontal cortex (polar frontal), accompanied by activation of the neocortical sensorimotor areas, as well as a deactivation of limbic structures^35^. Another study examining improvisation in rap musicians^36^ found, after spontaneous improvisation, dissociated activity in the medial and dorsolateral prefrontal cortices. Other investigations have explored changes in functional and structural connectivity during the learning and acquisition of new musical skills by means of training from an early age, finding changes in the motor network, including the corticospinal tracts^37^, pyramidal tracts^38^, the corpus callosum^39^, the internal capsule^40,41^ and the auditory-motor circuit^42^.

In the case of studies of deliberate or controlled creativity in musicians, various paradigms have been used, including improvising from a rhythmic structure^17,43,44^ or a melodic structure^45,46^; or both^43^; listening to a melody^47,48^; making tonal adjustments using keys and a set of tones^28^; or performing a specific musical creativity task based on the particular characteristics of the instrument of expertise of the participants, such as the piano^4,49^. The results of these studies have shown greater activity in the dorsolateral and inferior frontal cortex, the superior temporal gyrus, the supramarginal gyrus and the supplementary motor and premotor areas that are co-activated during any type of task, indicating the areas involved in auditory-sensorimotor integration^45^. In deliberate rhythm tasks comparing musicians and non-musicians, the prefrontal cortex is activated to a greater extent in musicians than non-musicians, while secondary motor regions were recruited to the same extent^44^. Another investigation of melodic improvisation and pulsation of pseudo-random keys in pianists has found greater activity of the bilateral inferior frontal gyrus, insula, anterior cingulate cortex, motor area (pre-SMA) and bilateral cerebellum^46^.

However, the few existing works that have employed deliberate musical creativity tasks appear to present a number of limitations. Firstly, most of the investigations on musical creativity using controlled tasks studied musicians who were performers of a specific musical style and instrument, usually piano and jazz. In general, they have found common findings about the role of the frontal lobe and the executive functions in the creative process^20^. And whilst such studies allowed for identifying the neural correlates of musical creativity associated with that instrument or style, they offer relatively little information on the cerebral regions involved in deliberate musical creativity in general. In our opinion, it is extremely important to study a range of different musical specialties in order to identify the specific areas that play an effective role in the deliberate musical creative process. Another significant weakness of studies in the current literature is that they have not explored the link between brain neuroimaging results and behavioural tests of creativity, intelligence, and musical improvisation in musicians and non-musicians. Studies considering these three dimensions could confirm whether these brain areas are related to deliberate musical creativity in neurocognitive terms.

On the basis of the above considerations, the main objective of this study was to investigate the brain activity, using fMRI, associated with a general task of deliberate musical improvisation -specifically rhythmic improvisation- in musicians with more than 10 years of musical training in different musical specialties/instruments, and in people without musical training, and to determine if these activations are linked to musical improvisation ability as well as scores on a general creativity test. On the basis of previous results reported in the literature^32^, we hypothesized that there would be greater cerebral activation in musicians compared with non-musicians, specifically in the prefrontal cortex and motor regions, as well as a greater deactivation of the temporoparietal junction. These results will extend the generality of previous findings in musicians playing their instrument of expertise to a more general situation with musicians trained in different instruments, performing a controlled musical creativity task such as a rhythm improvisation task. In addition, we hypothesized that in both groups brain activation during the task will correlate with performance on the musical improvisation task and the scores obtained on the creativity tests^49^.

## Results

### Behavioural results

The groups did not differ on any of the subscales of the creativity test (all p > 0.3). The musicians showed a significantly higher Intelligence Quotient (IQ) score than the non-musicians (p = 0.003) (see Table 1). The two groups did not differ in terms of precision when repeating the sequences in the repeat condition (p = 0.150). The musicians improvised for a longer time (p = 0.026) and played more notes during that time (p < 0.001) than the non-musicians. Finally, we calculated the Levenshtein edit distance between the Repeat and the Improvisation performance to estimate the extent to which the improvisation differed from the original rhythm. We found that musicians performed a rhythm that differed more from the original when compared with non-musicians (p < 0.001) (see Table 2).

**Table 1:** Behavioral data on creativity and IQ tests (means and standard deviations [SD]).

	Musicians	Non-musicians	p-value
Creativity Test			
General Creativity	133.32 (39.56)	124.86 (29.44)	0.445
Narrative Creativity	119.42 (38.22)	110.71 (29.24)	0.421
Fantasy	23.26 (9.97)	23.10 (10.03)	0.958
Narrative Fluency	53.68 (18.49)	48.76 (12.98)	0.332
Flexibility of thinking	37.63 (7.44)	36.62 (6.35)	0.645
Narrative Originality	28.63 (14.30)	25.33 (12.27)	0.437
Graphic Creativity	13.89 (4.60)	14.14 (4.68)	0.867
Graphic Originality	6.42 (3.50)	5.43 (2.86)	0.330
Elaboration of the response	1.89 (1.15)	2.29 (1.93)	0.447
Creative details	1.21 (1.03)	1.52 (0.93)	0.318
Title	4.37 (2.31)	4.90 (1.97)	0.434
K-Bit intelligence test			
Intelligence Quotient (IQ)	114.79 (4.45)	109.43 (5.90)	**0.003**

**Table 2:** Behavioral performance during fMRI task (means and standard deviations [SD]).

	Musicians	Non-musicians	p-value
Repetition accuracy (%)	97.91 (3.49)	96.12 (3.94)	0.150
Time spent improvising (sec)	7.88 (2.39)	6.36 (1.60)	**0.026**
Notes played while improvising	17.47 (6.51)	10.64 (2.44)	**<0.001**
Levenshtein edit distance between Repeat and Improvisation performance	8.94 (5.42)	3.89 (2.09)	**0.001**

The time spent improvising and the number of notes did not correlate with any of the scales of the creativity test.

### Neuroimaging results

In order to study the neural substrates of musical creativity, participants were first asked to repeat a rhythm previously heard, and then improvise a new one, including any modifications from the one they had just heard and repeated.

During the improvising condition, in comparison with reproducing, both groups activated the right dorsolateral prefrontal cortex and the supplementary motor area, extending activation to the anterior dorsal cingulate cortex. In particular, the musicians also bilaterally activated the superior frontal gyrus, the frontal operculum, the inferior parietal cortex, the anterior part of the insula and the cerebellum, as well as the left dorsolateral prefrontal cortex and the motor cortex (see Table 3).

**Table 3:** Brain regions showing significant within-group activations in the “Improvise > Repeat” contrast. ACC, Anterior Cingulate Cortex; BA, Brodmann Area; R, Right; L, Left; ^a,b^indicates part of the same cluster.

	BA	Side	MNI Coordinates			Cluster Size	Cluster p-value	t-value	Voxel p-value
			X	Y	Z				
**Musicians**									
Supplementary Motor Area	6, 8	R/L	4	14	62	7879^a^	<0.0001	9.28	<0.0001
Dorsal ACC	32	R/L	−4	24	42	7879^a^		8.50	<0.0001
Dorsolateral Prefrontal Cortex	9, 46	L	−42	36	24	7879^a^		7.67	<0.0001
Dorsolateral Prefrontal Cortex	9, 46	R	42	42	24	1006	<0.0001	6.38	<0.0001
Superior Frontal Gyrus		R	20	12	58	7879^a^		6.60	<0.0001
Superior Frontal Gyrus		L	−12	10	56	7879^a^		5.45	<0.0001
Frontal Operculum		L	−46	20	−2	7879^a^		6.51	<0.0001
Inferior Frontal Gyrus		L	−48	10	20	7879^a^		5.91	<0.0001
Anterior Insula	13	L	−32	22	6	7879^a^		6.22	<0.0001
Motor Cortex	6	L	−36	2	34	7879^a^		5.40	<0.0001
Inferior Parietal Cortex	40	R	46	−34	44	841	<0.0001	7.37	<0.0001
Inferior Parietal Cortex	40	L	−52	−40	48	899	<0.0001	7.00	<0.0001
Cerebellum		L	−34	−62	−28	879	<0.0001	6.41	<0.0001
Cerebellum		R	36	−52	−32	494	0.0004	6.01	<0.0001
Frontal Operculum		R	50	16	0	784^b^	<0.0001	5.37	<0.0001
Inferior Frontal Gyrus		R	50	14	14	784^b^		4.52	<0.0001
Anterior Insula	13	R	34	26	−2	784^b^		4.66	<0.0001
**Non**-**Musicians**									
Supplementary Motor Area	6, 8	R/L	10	26	62	752	<0.0001	5.52	<0.0001
Dorsolateral Prefrontal Cortex	9, 46	R	28	48	32	226	0.0098	4.80	<0.0001

ACC, Anterior Cingulate Cortex; BA, Brodmann Area; R, Right; L, Left; a,b indicates part of the same cluster. All results survived the Alphasim correction for multiple comparisons.

During the reproducing condition, in comparison with the improvising condition, both groups activated the occipital cortex, the parahippocampal and fusiform gyrus, and the bilateral hippocampus. Additionally, the musicians activated regions of the midline of the brain such as the precuneus, the medial prefrontal cortex, the subgenual anterior and posterior cingulate cortices as well as bilaterally the temporal cortices and the left angular gyrus (see Table 4).

**Table 4:** Brain regions showing significant within-group activations in the “Repeat > Improvise” contrast.

	BA	Side	MNI Coordinates			Cluster Size	Cluster p-value	t-value	Voxel p-value
			X	Y	Z				
**Musicians**									
Precuneus	31	R/L	−6	−62	22	20569^a^	<0.0001	9.58	<0.0001
Hippocampus		L	−32	−28	−12	20569^a^		9.71	<0.0001
Hippocampus		R	30	−10	−20	20569^a^		6.21	<0.0001
Fusiform Gyrus	37	L	−30	−36	−16	20569^a^		9.28	<0.0001
Fusiform Gyrus	37	R	34	−50	−10	20569^a^		6.84	<0.0001
Parahippocampal Gyrus		L	−24	−38	−10	20569^a^		8.85	<0.0001
Parahippocampal Gyrus		R	36	−34	−12	20569^a^		8.63	<0.0001
Occipital Cortex	19	R	44	−72	0	20569^a^		9.89	<0.0001
Occipital Cortex	19	L	−42	−76	6	20569^a^		7.13	<0.0001
Middle Temporal Cortex	21	L	−64	−8	−14	20569^a^		8.43	<0.0001
Angular Gyrus	39	L	−42	−54	24	20569^a^		7.97	<0.0001
Posterior Cingulate Cortex	31	R/L	−2	−48	34	20569^a^		7.39	<0.0001
Medial Prefrontal Cortex	10, 11	R/L	6	40	−12	1577^b^	<0.0001	7.04	<0.0001
Subgenual ACC		R/L	6	30	−10	1577^b^		5.39	<0.0001
Middle Temporal Cortex	21	R	56	−4	−16	757	<0.0001	6.79	<0.0001
**Non-Musicians**									
Occipital Cortex	19	R	44	−76	0	3915^c^	<0.0001	6.42	<0.0001
Occipital Cortex	19	L	−26	−80	16	4524^d^	<0.0001	6.14	<0.0001
Parahippocampal Gyrus		R	34	−36	−12	3915^c^		6.17	<0.0001
Parahippocampal Gyrus		L	−26	−40	−12	4524^d^		4.40	<0.0001
Fusiform Gyrus	37	R	34	−44	−12	3915^c^		6.15	<0.0001
Fusiform Gyrus	37	L	−40	−38	−14	4524^d^		5.55	<0.0001
Hippocampus		R	32	−22	−12	3915^c^		5.54	<0.0001
Hippocampus		L	−32	−26	−12	4524^d^		4.02	0.0001

ACC, Anterior Cingulate Cortex; BA, Brodmann Area; R, Right; L, Left; ^a,b,c,d^ indicates part of the same cluster. All results survived the Alphasim correction for multiple comparisons.

**Figure 1:**
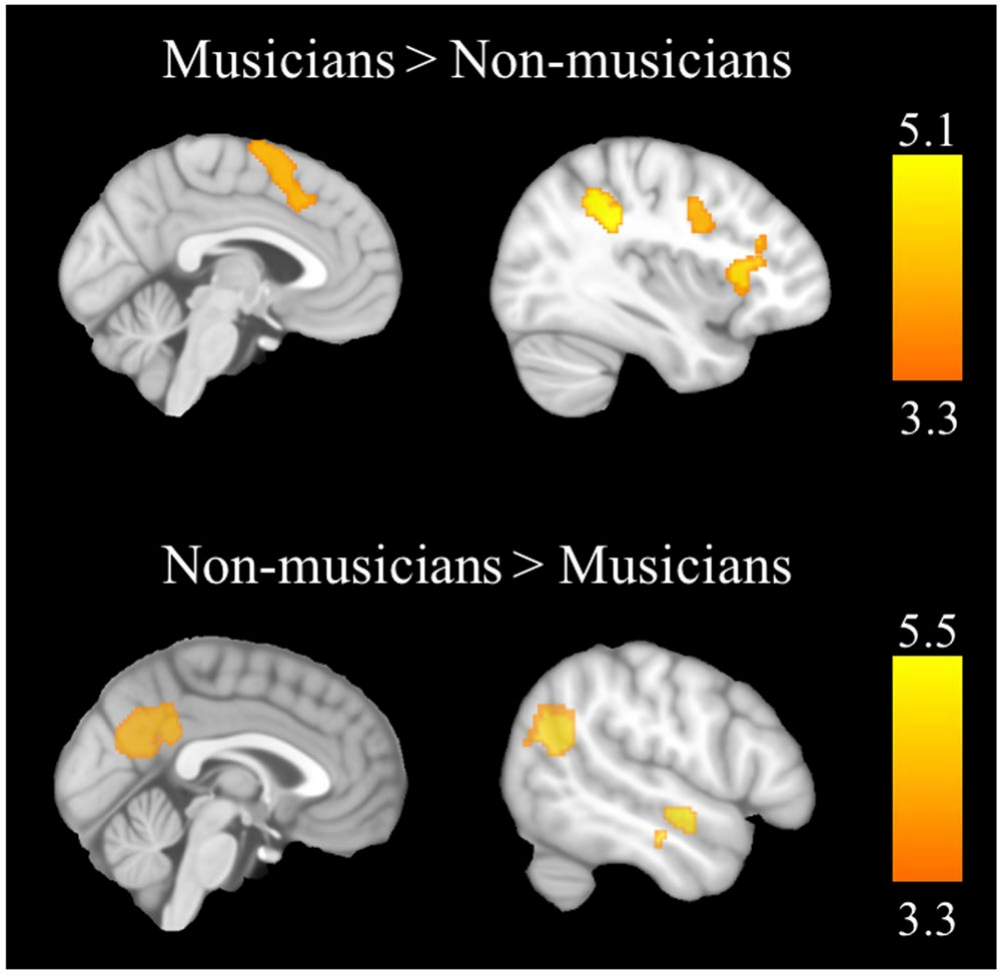
Brain regions showing significant between-group differences in the “Improvise > Reproduce” contrast. The colour bars and clusters indicate t-values.

The comparison between the two groups revealed that during the improvising condition, the musicians showed higher activation of the motor cortex and the supplementary motor area, the dorsal portion of the anterior cingulate cortex, and specifically in the left hemisphere, the dorsolateral prefrontal cortex, the inferior frontal gyrus, the frontal operculum, the anterior insula, and the inferior parietal cortex in comparison with the non-musicians (see Table 5, and Fig. 1). In contrast, during the improvising condition the musicians, in comparison with the non-musicians, showed greater deactivation of three regions of the DMN: the precuneus, the angular gyrus, and the left middle temporal cortex.

**Table 5:** Brain regions showing significant between-group differences in the “Improvise > Reproduce” contrast.

	BA	Side	MNI Coordinates			Cluster Size	Cluster p-value	t-value	Voxel p-value
			X	Y	Z				
**Musicians > Non-Musicians**									
Supplementary Motor Area	6, 8	R/L	4	12	62	1300^a^	<0.0001	5.08	<0.0001
Motor Cortex		L	−38	2	34	1300^a^		4.47	<0.0001
Dorsal ACC	32	R/L	−8	22	38	1300^a^		3.64	0.0004
Inferior Frontal Gyrus	44	L	−50	10	18	1300^a^		4.22	<0.0001
Frontal Operculum	47	L	−48	18	0	344^b^	0. 0022	4.27	<0.0001
Anterior Insula	13	L	−30	22	10	344^b^		4.66	<0.0001
Dorsolateral Prefrontal Cortex		L	−42	32	24	344^b^		3.90	0.0002
Inferior Parietal Cortex	40	L	−52	−40	46	386	0.0013	4.76	<0.0001
**Non**-**Musicians > Musicians**									
Precuneus/Posterior Cingulate	31	R/L	−4	−66	24	984	<0.0001	5.42	<0.0001
Middle Temporal Cortex	21	L	−64	−8	−14	297	0.0039	5.39	<0.0001
Angular Gyrus	39	L	−42	−54	24	640	<0.0001	5.13	<0.0001

ACC, Anterior Cingulate Cortex; BA, Brodmann Area; R, Right; L, Left; ^a,b^ indicates part of the same cluster. All results survived the Alphasim correction for multiple comparisons.

### Correlations

#### Neuroimaging task

**Figure 2:**
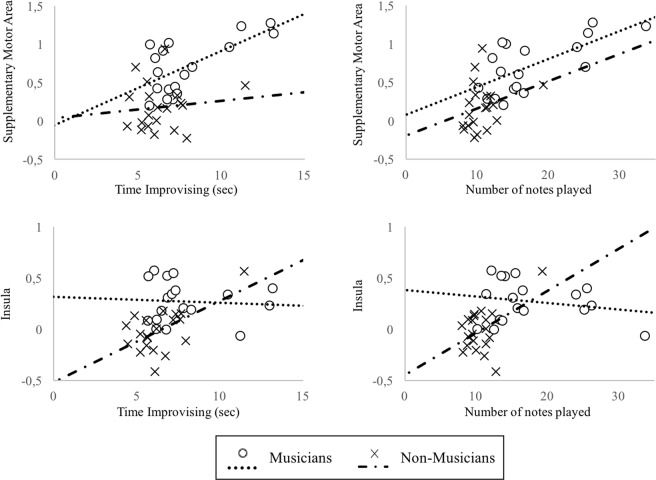
Correlations between SMA and Insula activation and the time and the number of notes played during improvising. Dots and dots line represent musicians, crosses and dash dotted line represent non-musicians.

In the group of musicians, the time spent improvising and the number of notes played correlated with the activation of the supplementary motor area (r = 0.657, p = 0.003 and r = 0.678, p = 0.002, respectively), while in the group of non-musicians this correlation was not significant (r = 0.117, p = 0.624 and r = 0.283, p = 0.227) (see Fig. 2).

In contrast, in the group of non-musicians it was found that the time spent improvising and the number of notes played correlated with the activity of the anterior insula (r = 0.603, p = 0.005 and r = 0.478, p = 0.033), whereas in the group of musicians no such correlations were found (r = −0.069, p = 0.787 and r = −0.204, p = 0.416) (see Fig. 2).

#### Creativity task

No significant correlations were found between brain activations during the fMRI task and the total score on the creativity test for either the whole sample or each of the groups.

## Discussion

The aim of the present study was to investigate and compare the cerebral mechanisms underpinning deliberate musical creativity in musicians and non-musicians, as well as to explore the links between brain activity and creative thinking and (controlled) musical behaviour. The results lend support to our hypothesis that musicians —in comparison with non-musicians — show greater activation of different motor regions (e.g., the supplementary area and the motor cortex), left prefrontal areas (inferior frontal and dorsolateral cortices), as well as the insula and the inferior parietal cortex. Further, in musicians we found a greater deactivation of the default brain network, in line with the expected deactivation of the temporoparietal junction. In the case of the musicians a correlation was found between the amount of time spent improvising and the activation of the supplementary motor area; however, in the non-musicians, improvisation time was associated with activation of the insula. Contrary to our expectations, no significant correlations were found between brain activation and scores on the general creativity test.

Our results for both groups regarding brain activation during the musical improvisation tasks replicate the activation patterns found in previous studies on musical creativity^32^. The fact that areas previously linked to musical creativity were activated indicates the effectiveness of the methodology employed in the present study, since in both groups the deliberate control creativity task activates brain structures commonly seen in the process of creativity^20,26^. Most of the brain regions involved in musical creativity also interact in other processes, which indicates that creative thinking is based on distributed networks^46^. In this way, the areas activated in the brain of our volunteers while improvising (i.e. the dorsolateral prefrontal cortex)^20,50^ and the anterior dorsal cingulate cortex^27^ are the areas typically activated during creativity^1,51^. These involve mental displacement for the planning of action by means of the search for information and selection of novel ideas of relevance; combining elements in semantic networks^20,29^; inhibitory control and updating of working memory^28^; and collaboration in the analytical and deliberate processing of information, assigning value to novel ideas^29^. The other areas most activated in musicians (and commonly associated with motor behaviour and sound processing) are the supplementary motor cortex^1^, as well as the dorsolateral prefrontal cortex^32^. In creative terms, the supplementary motor area is also involved in the creative process of improvisation^3,46^. In addition, some studies have reported greater activation of these same auditory-sensory-motor areas in musicians in comparison with non-musicians^45^.

Other areas found to be more active in musicians than in non-musicians in our study — such as the left inferior frontal gyrus or the inferior parietal cortex — have also been linked to both rhythmic and melodic creative processes^52^. Finally, we also observed greater activation of the anterior insula in the group of musicians. This region is associated with the dorsal anterior cingulate cortex for monitoring and detecting relevant behavioural stimuli, and is also involved in the alternating dynamics between the previously mentioned networks^7,15,24,53^.

With regard to the greater deactivation of the Default Mode Network (DMN) in musicians, it is known that the completion of a cognitive task requires the activation of regions dedicated to attentional focus, reasoning skills, and working memory, all of which are directed towards the task, solving the problem by allocating cognitive resources to meet specific objectives^54^. It is also known that when activation occurs in order to solve the task, there is simultaneous deactivation of the DMN. It is therefore unsurprising that the greater activation of all the aforementioned regions dedicated to the performance of the task is accompanied by a greater deactivation of the DMN in our group of musicians.

When conducting the comparisons between groups, we observed that there were no significant behavioural differences in terms of performance in the musical reproduction condition. However, differences were observed when improvising, with the finding that musicians spent more time improvising, played a higher number of notes and created new rhythms that differed more from the original, which is unsurprising, given their greater experience in the field of music. However, to our surprise, in our sample of musicians a correlation was found between improvisation time and activation of the supplementary motor area, whilst in non-musicians, improvisation time was found to correlate with activation of the insula. According to the literature, the insular zone is related to the unification of multisensory information^55,56^, integrating and maintaining the balance of internal and external information^55^ and, in addition, it coordinates brain networks involved in affective processes and executive order^56^, as well as musical performance^57^. This zone is related to guiding external attention, self-related cognition^56^, interoceptive awareness^58^ and the activation of motor sensory information^59^. In addition, the insular cortex plays an essential role in emotional processing, and is involved in creative thinking, allowing the executive network and DMN to notice emotionally promising counterfactual elements in the environment and associations in the mind^60^. Since the anterior insula plays a central in the relevant network, this becomes important when we switch from a conventional way of thinking to a new perspective^61^.

In contrast, for our group of musicians, activation of the supplementary motor area (SMA) correlated with improvisation time. This area is activated in tasks that require motor programming and execution^62^, participating in cognitive control^63^, in the planning of complex motor movements, as well as during listening and musical performance^62^. However, the SMA is also involved in sensorimotor representation^62^ and in the processing of sequences in several cognitive domains, such as action sequences, time processing, spatial processing, numerical cognition, perception of language, and music and production^64,65^. The fact that this region plays a crucial role in general domain sequential processes — contributing to the integration of sequential elements in higher order representations regardless of the nature of those elements — and is essential to musical performance, is compatible with the results of previous studies suggesting that this region plays a central role in music processing^64^. We can therefore suppose that in the group of musicians — who have a presumed theoretical/practical background in music and are consequently gifted in the subject — the supplementary motor area constructs the internal representation of musical performance and processing, integrating the multimodal information required for performance^62^, adequately planning the required range of complex motions. However, for the non-musicians — who have not had such experience —their improvisational behaviour is guided by the insula, favouring the composition of multisensory information in musical performance. Thus, for non-musicians, by integrating the sensory data of the acquired information (the task), which is perceived as novel and relevant from their perspective, the relevance network and the insula are activated by the stimulation of improvisational behaviour. In addition, our results suggest that non-musicians, since they do not have cognitive musical experience, engage emotional processes in order to construct creative thoughts about the task, switching from the habitual problem-solving mode to a new way of thinking.

Another important finding in our study was the absence of correlations between the PIC-A general creativity score and both brain activity and performance on the magnetic resonance task. The absence of significant correlations seems to indicate that musical creativity — both from a cerebral and behavioural point of view — is specific to the musical field, and is not related to creativity capacities in other more general domains. Future research should explore whether the capacity for musical creativity can be associated with other more specific creative fields.

This study has several strengths. Firstly, from a neuroimaging point of view the methodology employed here is robust, adopting a previously validated task and using statistical thresholds corrected by multiple comparisons. Moreover, our sample of musicians has an extensive background of training and musical experience, allowing us to study a population that is expert in a task specifically designed to measure musical creativity. Further, to the best of our knowledge, this is the first study to explore the relationship between creativity associated with a specific field (in this case, music) and general creativity using a widely used instrument such as the PIC-A. Additionally we confirmed that all participants performed the task properly. We checked their performance both during practice and the scanning session and verified that they repeated the original rhythm with a high level of accuracy and made substantial changes during the improvisation. Finally, the presence of musicians with various specialties and the use of a rhythmic task allows for generalization of the results that was not possible in previous studies since all the participants were from the same specialty and performed a task specific to that instrument.

However, there are also a series of limitations that must be taken into account. First, whilst our study groups are of sufficient size, they are still limited in number. Future studies should replicate these results with larger samples. In addition, there were differences between the two groups in terms of IQ scores, although we took steps to control for the effects of this variable by including it as a covariate in all statistical analyses.

In summary, our study has revealed that musicians, in comparison with non-musicians, showed higher activation of different motor regions, left pre-frontal areas, the insular cortex, and the inferior parietal region whilst at the same time showed greater deactivation of the DMN areas. In addition, the brain areas related to musical improvisation time appeared to differ according to musical experience. In the case of musicians, a correlation was found between the improvisation time and activation of the supplementary motor area. However, in the non-musicians, improvisation time was associated with activation of the insula. Future studies should aim to replicate these findings in larger samples with a wider variety of instruments and investigate in more depth the relationships between the brain areas found and the various parameters of musical behaviour.

## Methods

### Participants

Sample size was estimated based on a recent study on brain differences between musicians and non-musicians, which reported a Cohen’s d of 2^57^. Therefore, to obtain a statistical power of 0.8, with an alpha level = 0.05, the minimum sample required was 13 participants per group, according to the recommendations of Zandbelt^66^ for voxel-based analyses. We included an additional 50% of participants to avoid the potential effects of dropout.

Our sample of participants was composed of 21 musicians (11 women and 10 men) with at least 10 years of musical experience (see Table 6) and 21 non-musicians (5 men and 16 women) aged between 17 and 26 years. The inclusion criterion for the group of musicians was to have more than 10 years of formal training in music (mean = 14.19 years, SD = 2.58), while participants in the control group were required to have no experience in musical training beyond compulsory studies in school. Exclusion criteria were the presence of any type of serious acoustic or medical problem, having suffered head injuries with loss of consciousness for more than 30 minutes, or the consumption of drugs, all recorded by means of a personal interview. In addition, participants must not have suffered damage or psychopathological dysfunctions, as measured using the Symptom Checklist-90-R. Any incompatibility issues with the magnetic resonance session (e.g., pregnancy, claustrophobia, or the presence of ferromagnetic implants) were also considered exclusion criteria. All participants had normal hearing ability and normal or corrected vision.

**Table 6:** Demographic data & musical specialty of the participants.

Musicians				Non-musicians		
Code	Sex	Age	Musical specialty	Code	Sex	Age
M01	Male	21	Guitar	NM01	Male	18
M03	Male	24	Tuba	NM02	Female	22
M04	Female	21	Clarinet	NM03	Female	23
M05	Male	23	Piano & guitar	NM04	Female	20
M06	Male	19	Piano	NM05	Male	18
M07	Female	19	Guitar	NM06	Female	18
M08	Male	20	Guitar	NM07	Female	19
M09	Female	18	Singing	NM08	Female	21
M10	Female	19	Piano	NM09	Female	22
M11	Male	22	Clarinet	NM10	Female	26
M12	Male	19	Piano	NM11	Female	19
M14	Female	20	Bassoon	NM12	Female	18
M15	Male	18	Guitar	NM13	Female	20
M16	Female	18	Piano	NM14	Male	24
M17	Female	17	Piano	NM15	Male	18
M18	Female	20	Guitar	NM16	Female	21
M19	Female	21	Clarinet	NM17	Male	19
M20	Female	22	Piano &Singing	NM18	Female	18
M21	Male	24	Guitar	NM19	Female	18
				NM20	Female	19
				NM21	Female	23

Two participants from the group of musicians were excluded due to excessive movement (>2 mm) during the fMRI procedure (explained in the section titled ‘acquisition and preprocessing of imaging data’). Therefore, the final sample consisted of a group of 19 musicians with an average age of 20.26 years (SD = 2.05), 10 of which were women (52.6%) and 9 men (47.4%), as well as a group of 21 non-musicians with a mean age of 20.19 years (SD = 2.36), 16 of which were women (76.2%) and 5 (23.8%) of which were men (see Table 6). The groups did not differ significantly in terms of sex (p = 0.119), age (p = 0.918), or education level (p = 0.199).

The recruitment process was carried out randomly at the University of Granada. This study was approved by the ethical committee for human research at the University of Granada and was conducted in accordance with the Helsinki declaration. All participants were duly informed of the investigation and gave their signed informed consent, confirming their voluntary participation in the study.

### Procedure

The research was carried out in two one-hour sessions. First, we assessed the performance of the participants on neurocognitive tests of creativity and intelligence. Subsequently the task of musical creativity was carried out inside a magnetic resonance imaging scanner. Both sessions took place at the Mind, Brain, and Behavior Research Center (CIMCYC) of the University of Granada.

### Instruments

The performance of the participants on neurocognitive tests of imagination and creativity was assessed using the Creativity Imagination Test (PIC-A)^67^ and the Kaufman Brief Intelligence Test (K-BIT)^68^.

#### Creativity Imagination Test (PIC-A)

The creativity test used in this research was the Creativity Imagination Test (PIC-A) which measures creativity through the use of imagination. The PIC-A considers several variables that have been shown to be relevant for the study of creativity: Fantasy, Fluency of ideas, Flexibility of thinking, Originality of the responses, Elaboration of the responses, use of Creative Details such as color, shadow and expansiveness and Title. It consists of four tests, the first three evaluate verbal or narrative creativity and the last one evaluates graphic creativity.

The first test involves observing a drawing, and then imagining and writing everything that could be happening in that scene. This allows for triggering the imagination and fantasy processes and exploring the ability to formulate hypotheses and think in terms of what is possible. The second test is an adaptation of the Guilford Test “Uses of a Brick”. It consists of making a list of all the things for which a certain object could be useful. This part evaluates the ability to “redefine” problems: that is, the ability to find uses, functions and applications different from the usual ones, to speed up the mind and to offer new interpretations or meanings to familiar objects to give them a new use or meaning. The third test presents an improbable situation to the participants, after which they are required to say what they think would happen if it were true (e.g., “Imagine what would happen if we never stopped growing”). It evaluates the capacity to fantasize and the ability to handle unconventional ideas that the participant would probably not dare to express in more serious situations, as well as assessing openness and receptivity when faced with novel situations. Finally, the fourth test of graphic imagination is inspired by items from the Torrance test. It consists of completing drawings from some given strokes, and giving each one a title in a creative way. This test discriminates subjects who have few ideas but who work a lot, with great imagination, from those subjects who have very original ideas but have difficulty elaborating them.

The scoring system is relatively easy and well explained in the manual. The ratings for Fantasy, Flexibility of thinking, Narrative Fluency and Narrative Originality were obtained from the first three tests, along with a global score of Narrative creativity. From the fourth test we obtained the ratings of Graphic Originality, Elaboration of the responses, Creative Details, Title, and a global score of Graphic creativity. At the end, a final score of General Creativity was calculated as the sum of the narrative and graphic global ratings. The psychometric evaluation of the PIC-A showed internal consistency with a Cronbach’s Alpha of 0.85, whilst construct validity was in accordance with the theory^67^.

#### The Kaufman Brief Intelligence Test (K-BIT)

The Kaufman Brief Intelligence Test (K-BIT) was used to assess the intelligence of the participants. The K-BIT measures cognitive functions through two tests: verbal (vocabulary, composed of two tests), and nonverbal (matrix), which evaluates crystallized and fluid intelligence, and obtains a compound Intelligence quotient (IQ). This test could be used in people from 4 to 90 years.

The verbal test evaluates the knowledge of words and verbal concept formation, whilst the nonverbal part measures fluid intelligence and the participants’ ability to solve new problems by perceiving relationships and completing analogies. The raw scores from each test were converted into typical scores with a mean of 100 and standard deviation of 15. The Spanish version of the K-BIT presented a test-retest reliability coefficient resulting from the correlations found for vocabulary (0.94) and for matrices (0.86), whilst internal consistency for the compound score was evidenced by a value of 0.90^68^.

### fMRI task

To evaluate the brain response associated with musical creativity, a musical improvisation task was used during a functional magnetic resonance session. We used a modified version of the musical creativity task developed by Bengtsson *et al*.^17^. In that study they examined the neural substrates of improvisation in pianists, so they instructed their participants to perform and memorize an improvisation (experimental condition), and repeat it afterwards (control condition). Conversely, given that we were interested in comparing the improvisation processes in musicians and non-musicians, that task would be difficult for people not trained in playing music, and they would probably fail to memorize and repeat the improvisation. Accordingly, we changed the conditions of the experiment and our participants were instead asked to repeat a rhythm (control condition) and then perform an improvisation (experimental condition). Participants were asked to lie down on the scanner and they were instructed to press with their fingers an Evoke Response Pad System (Resonance Technology Inc., Northridge, California) in the same way that they press the key of a piano or beat a drum.

All participants completed three conditions during the task: Improvise, Repeat, and Rest. Initially, they were prompted with the name of the condition during the first two seconds of each trial. For the Rest condition, the screen went black for the following 21 seconds and participants were instructed to keep still and relax. For the other conditions a rhythm score appeared on the screen for 7 seconds. The score was enclosed within a red, rectangular frame and participants listened to the rhythm through magnetic resonance-compatible earplugs. The frame then disappeared and for the remaining 14 seconds they were required to follow the instructions shown previously. During the Improvise condition the instruction was to play a new rhythm employing any kind of modification of the presented rhythm score. In the Repeat condition participants were required to reproduce the rhythm that they had just heard.

**Figure 3:**
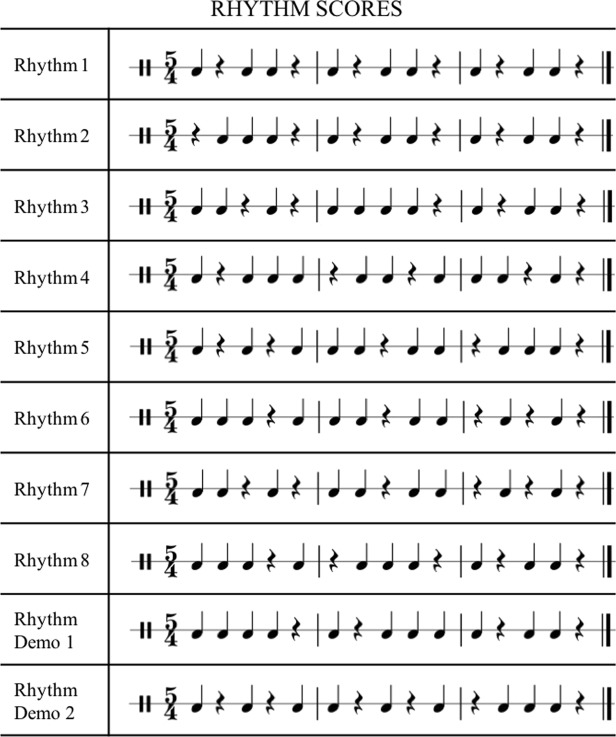
Scores of the rhythms used in the fMRI task.

A total of 8 rhythm scores (Fig. 3) were used in the experiment. They were written for the present study. The order of the conditions was fixed and they always performed the Improvise condition before the Repeat condition to favour improvisation when they have heard the rhythm just once. The total time taken to complete the task was 9 minutes and 12 seconds. The total time and number of keys played during the Improvise condition were recorded. We also recorded the time and number of keys played during the Repeat condition in order to determine how accurately they repeated the rhythms. Finally, the Levenshtein edit distance between the Repeat and the Improvisation performance was calculated to estimate the extent to which the improvisation differed from the original rhythm.

The task was administered using the Presentation software (version 1.8; http://neurobs.com) and displayed on a resonance-compatible screen through an inverted mirror system. Prior to the MRI session, all participants were trained on the task. To ensure that they understood the task and were able to perform it, the researchers carefully explained to the participants that improvisation means any change in the rhythm that they have just heard, for example changing one position of a pause or changing the speed of the beats. They completed each condition two times using different rhythms to those heard when inside the scanner, during which researchers checked that they actually performed the improvisation.

### Acquisition and preprocessing of imaging data

Magnetic resonance images were acquired on a 3 Tesla Magnetom Tim Trio scanner (Siemens Medical Solutions, Erlangen, Germany) equipped with a 32-channel receive-only head coil. During task performance, a T2*-weighted echo-planar imaging (EPI) sequence was acquired with the following parameters: Repetition time (TR): 2000 ms; echo time (TE): 25 ms; flip angle: 80°; field of view (FOV): 238 mm; number of slices: 35; voxel size: 3.5 × 3.5 × 3.5 mm; gap: 0.7 mm; number of volumes: 276. Images were collected axially and parallel to the AC-PC plane. In the same session, a sagittal three-dimensional T1-weighted image was also obtained for anatomical reference and to discard gross anatomical abnormalities. The parameters were as follows: TR: 2300 ms; TE: 3.1 ms; flip angle: 9°; FOV: 256 mm; number of slices: 208; voxel size: 0.8 × 0.8 × 0.8 mm.

Functional images were preprocessed using the Statistical Parametric Mapping (SPM12) software (Wellcome Department of Cognitive Neurology, Institute of Neurology, Queen Square, London) running under Matlab R2017 (MathWorks, Natick, MA, USA). Preprocessing included re-slicing to the first image of the time series, unwarping, coregistration with the structural image of each participant, normalization to an EPI template in the Montreal Neurobiological Institute (MNI) space, and spatial smoothing by convolution with a 3D Gaussian kernel [full width at half maximum (FWHM) = 8 mm]. Data from two musicians (M02 & M13) were discarded due to excessive movement (>2 mm) during the fMRI task.

### Statistical analyses

#### Behavioural analyses

Behavioural data were analysed with the Statistical Package for the Social Sciences version 20 (SPSS; Chicago, IL). We conducted independent sample t-tests (two-tailed) to compare groups in demographic, creativity, IQ, and fMRI task-related variables. All behavioural data followed a normal distribution as assessed with Kolmogorov-Smirnov tests (all p > 0.05).

#### Neuroimaging analyses

Three task regressors (Improvise, Repeat, and Rest) were modelled for the 14 seconds that participants had to follow the instructions and convolved with the SPM12 canonical hemodynamic response function. To prevent motion artefacts, six head motion parameters were entered as regressors of no interest in all first-level analyses. According to the aims of the study, we defined two contrasts of interest (i) Improvise > Repeat and (ii) Repeat > Improvise. Data were high-pass filtered to remove low frequency noise (1/128 Hz) and corrected for temporal autocorrelation using an autoregressive AR model.

One-sample t-tests were conducted on the resulting first-level contrast images to assess across-group activations in each contrast. Next, we conducted a two-sample t-test to assess between-group differences using the same first-level contrast images. To exclude potential confounds linked to IQ, this variable was included as a covariate in all analyses. In order to focus on the brain substrates of musical creativity we also included the General creativity scores as a covariate in all analyses. Both covariates were orthogonalized before being included in the models.

The statistical significance threshold was corrected for multiple comparisons using a combination of voxel intensity and cluster-extent thresholds. The spatial extent threshold was determined by 1,000 Monte Carlo simulations, using the AlphaSim algorithm as implemented in the SPM REST toolbox. Input parameters included a brain mask of 176 588 voxels, an individual voxel threshold probability of 0.001 and a cluster connection radius of 5 mm, considering the actual smoothness of data after model estimation. A cluster-extent threshold of 201 voxels was estimated.

To examine the association between brain activations and task performance in musicians and non-musicians, we conducted Pearson correlation analyses in SPSS. The beta eigenvalues from each peak of significant between-group differences in the Improvise > Repeat contrasts were extracted using a sphere of 5 mm and correlated with the behavioural task measures (i.e. total number of keys played and total time improvising) and the total score of the creativity test. To explore whether these relations are specific to each group, we conducted these analyses within each group.

## Data Availability

The datasets generated during and/or analysed during the current study are available from the corresponding author on reasonable request.
